# Heme-Mediated Induction of CXCL10 and Depletion of CD34+ Progenitor Cells Is Toll-Like Receptor 4 Dependent

**DOI:** 10.1371/journal.pone.0142328

**Published:** 2015-11-10

**Authors:** Carmen M. Dickinson-Copeland, Nana O. Wilson, Mingli Liu, Adel Driss, Hassana Salifu, Andrew A. Adjei, Michael Wilson, Ben Gyan, Daniel Oduro, Kingsley Badu, Felix Botchway, Winston Anderson, Vincent Bond, Methode Bacanamwo, Shailesh Singh, Jonathan K. Stiles

**Affiliations:** 1 Department of Biochemistry & Immunology, Morehouse School of Medicine, Atlanta, Georgia, United States of America; 2 Department of Pathology, Korle-Bu Teaching Hospital, University of Ghana Medical School, Accra, Ghana; 3 Department of Parasitology, Noguchi Memorial Institute for Medical Research, University of Ghana, Accra, Ghana; 4 Department of Immunology, Noguchi Memorial Institute for Medical Research, University of Ghana, Accra, Ghana; 5 Department of Biology, Howard University, Washington, DC, United States of America; 6 Cardiovascular Research Institute, Morehouse School of Medicine, Atlanta, Georgia, United States of America; Centro Cardiologico Monzino, ITALY

## Abstract

*Plasmodium falciparum* infection can cause microvascular dysfunction, cerebral encephalopathy and death if untreated. We have previously shown that high concentrations of free heme, and C-X-C motif chemokine 10 (CXCL10) in sera of malaria patients induce apoptosis in microvascular endothelial and neuronal cells contributing to vascular dysfunction, blood-brain barrier (BBB) damage and mortality. Endothelial progenitor cells (EPC) are microvascular endothelial cell precursors partly responsible for repair and regeneration of damaged BBB endothelium. Studies have shown that EPC’s are depleted in severe malaria patients, but the mechanisms mediating this phenomenon are unknown. Toll-like receptors recognize a wide variety of pathogen-associated molecular patterns generated by pathogens such as bacteria and parasites. We tested the hypothesis that EPC depletion during malaria pathogenesis is a function of heme-induced apoptosis mediated by CXCL10 induction and toll-like receptor (TLR) activation. Heme and CXCL10 concentrations in plasma obtained from malaria patients were elevated compared with non-malaria subjects. EPC numbers were significantly decreased in malaria patients (*P* < 0.02) and TLR4 expression was significantly elevated *in vivo*. These findings were confirmed in EPC precursors *in vitro*; where it was determined that heme-induced apoptosis and CXCL10 expression was TLR4-mediated. We conclude that increased serum heme mediates depletion of EPC during malaria pathogenesis.

## Introduction

*Plasmodium falciparum* infections are responsible for about 283 million malaria cases and 584,000 deaths annually, primarily in Sub Saharan Africa [1]. Approximately 30% of malaria related deaths occur in children under five years of age despite appropriate treatment, and it is estimated that a child dies from malaria complications every minute [2, 3]. Current malaria treatments target malaria parasite but offer limited protection to a subset (10–30%) of patients who die from severe malaria complications [4, 5]. Adjunctive therapies are urgently needed to offset these unacceptably high mortality rates.

**Fig 1:**
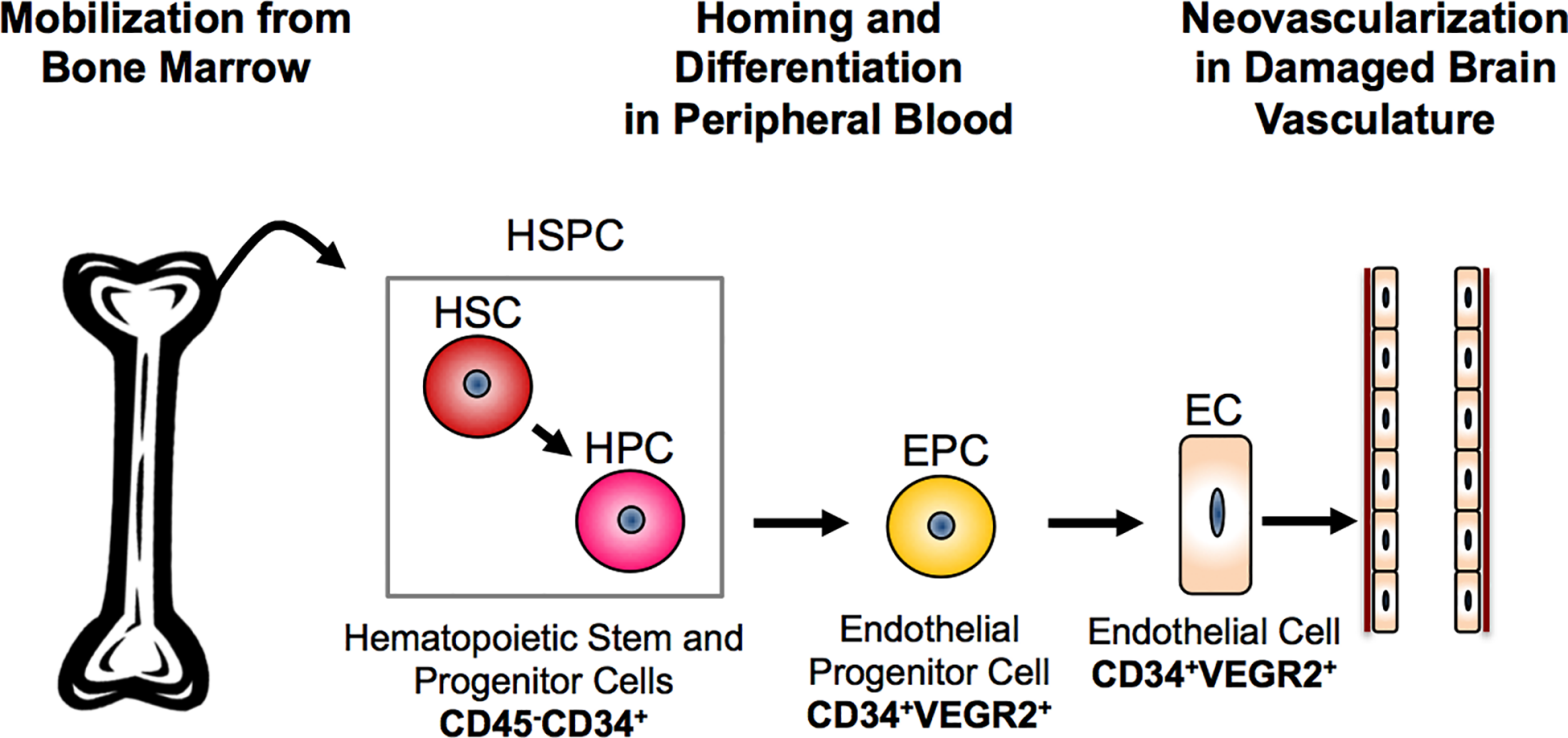
Hematopoietic Stem and Progenitor Cell Populations (HSPC) are vital to vascular endothelial repair and regeneration. HSPC are CD34^+^ cells derived from the bone marrow, where they reside in the stromal layer until mobilized in response to chemokines and cytokines released from dysfunctional endothelium. In the peripheral blood, they are capable of differentiating into endothelial progenitor cells (EPC) that will home into cites of vascular dysfunction. The EPC retains the hematopoietic surface marker CD34 in addition to gaining the vascular endothelial cell surface marker CD309 or VEGFR2. These cells will differentiate into mature and circulating endothelial cells capable of incorporating into sites of compromised vasculature, and inducing neovascularization as well as proliferation of existing endothelial cells.

Malaria mortality is associated with exaggerated host responses to inflammatory factors such as interferon gamma (IFNγ), tumor necrosis factor alpha (TNFα), free heme, C-X-C motif chemokine 10 (CXCL10) and parasite-derived cytotoxins [6–11]. Extensive hemolysis and increased plasma heme leads to vascular activation, inflammation and over production of CXCL10, which exacerbates the disease [8, 12, 13]. Previous studies indicate that increased serum levels of free heme and CXCL10 limited the ability of the host to repair and regenerate damaged blood-brain barrier (BBB) components during development of severe malaria pathogenesis and were predictive of poor prognosis of severe malaria [14]. In addition, studies indicate that endothelial progenitor cell (EPC) depletion and Toll-like receptors (TLR) 4 and 9 play an important role in malaria prognosis. EPCs and EPC-precursors are hematopoietic stem and progenitor cells expressing cluster of differentiation 34 (CD34). CD34 is a hematopoietic progenitor cell antigen associated with cell-cell adhesion and stem cell attachment, and a subset of CD34^+^ cells is capable of differentiating into microvascular endothelial cells (Fig 1) [15–18]. CD34^+^ hematopoietic stem and progenitor cells (CD34^+^-HSPC) are also blood-cell precursors of T- and B-lymphocytes, which are potently activated by microvascular damage and alterations in chemokine/cytokine expression [19–21]. In 2014, Belcher et al. found that heme-induced cytotoxicity involves the TLR4 signaling pathway in sickle cell disease, and may or may not be different than lipopolysaccharide-mediated TLR4 signaling [22, 23]. By-products of this signaling pathway result in increased expression of the heme-degrading enzyme, heme-oxygenase-1 (HO-1), CXCL10 and adhesion molecules such as vascular and intercellular cell adhesion molecules [12, 23, 24].

Recent reports have associated decreased circulating EPC with poor prognosis of severe malaria [25]. Understanding the mechanism involved in EPC depletion in malaria pathogenesis may provide a basis for development of therapies that would protect and retain the EPC function during malaria treatment or management.

The objective of this study was to determine the effects of free heme on EPC, characterized as CD45^-^CD34^+^VEGFR2^+^ cells *in vivo*, and the EPC precursor population, CD34^+^ hematopoietic stem and progenitor cells, characterized as CD34^+^-HSPC *in vitro*. CXCL10 and TLR4 expression in these cells were assessed after exposure to heme at different, physiologically relevant concentrations (10–60 μM). We hypothesized that CD34^+^-HSPC depletion during malaria pathogenesis is a function of heme-induced apoptosis mediated by induction of CXCL10 and TLR activation. Here, we report that free heme activates TLR4 expression and induces over production of CXCL10 resulting in apoptosis and decreased bioavailability of EPC and HSPC precursors.

## Materials

### Reagents and Antibodies

Heme was purchased from Frontier Scientific (Logan, UT). Camptothecin (CPT) and Lipopolysaccharide (LPS) were purchased from Sigma-Aldrich (St. Louis, MS), TLR4 inhibitor/antagonists monoclonal anti-CD14 antibody and Ethyl (6R)-6-[N-(2-chloro-4-fluorophenyl) sulfamoyl] cyclohex-1-ene-1 -carboxylate, (Takeda, TAK-242) were purchased from InvivoGen (San Diego, CA), 3-(4,5-dimethylthiazol-2-yl)-2,5-diphenyltetrazolium bromide (MTT) was purchased from Life Technologies (Grand Island, NY).

### Cell Lines

Human brain microvascular endothelial cell line (HBVEC; Biowhittaker, Walkersville, MD) was graciously provided by Dr. Bond’s laboratory at Morehouse School of Medicine. Their characteristics include expression of endothelial lineage markers VEGFR2, von Willebrand factor, CXCL10 and corresponding receptor CXCR3. Primary human CD34^+^ hematopoietic stem and progenitor cells (CD34^+^-HSPC) isolated from human bone marrow obtained from StemCell Technologies, Vancouver, Canada.

## Methods

### Ethical considerations

All study subjects were enrolled after written informed consent was obtained from them or their guardians. Informed consent and human subject research guidelines of the National Institutes of Health (NIH), and the Centers for Disease Control and Prevention (CDC) in the United States were followed. The IRB committees at Morehouse School of Medicine (USA) and the University of Ghana approved this study.

### Study sites and population

The study participants were recruited from the Greater Accra region which accounts for 4% of all malaria cases among children under 5 years and 27% of all outpatient department (OPD) malaria cases in Ghana [26]. The study samples were obtained from two study sites: Korle-Bu Teaching Hospital (KBTH) and the Shai-Osudoku District Hospital (SODH). KBTH is the leading referral and teaching hospital of the University of Ghana Medical School, which serves patients from diverse communities in the country. SODH is a district hospital serving the Shai-Osudoku District in Southeastern Ghana. Malaria is endemic and perennial in Ghana, with seasonal variations that are more pronounced in the northern region [26]. Malaria is the number one cause of morbidity and mortality in the country, accounting for approximately 38% of all OPD attendance, 36% of all admissions, and 33.4% of all mortality in children less than five years of age [27]. *P*. *falciparum* is the most prevalent in the country with occasional mixed infection with *P*. *malariae* [26].

### Enrollment criteria

#### Malaria patients

Malaria patients with both confirmed thick film slides and *Plasmodium* Lactate Dehydrogenase/Histidine Rich Protein-2 (pLDH/HRP-2) Antigen Combo Card rapid diagnostic test (RDT; BestNet, London, UK) were recruited into the study after informed consent. Parasitemia was evaluated microscopically on the number of parasites per field (+, 1–10 parasites/100 fields, ++, > 10 parasites/100 fields, +++, 1–10 parasites/field, and ++++, > 10 parasites/field) and at least 100 fields/slide were examined to rule out any negative thick film slide. Enrollees in this group had no evidence of impaired consciousness, seizures, past history of mental illness, meningitis or head injury.

#### Non-malaria subjects

Individuals with negative pLDH/HRP-2 RDT and no *P*. *falciparum* parasitemia were recruited and classified as non-malaria subjects.

Relevant data relating to age, sex, complete blood counts and available medical history were obtained from medical records as well as a survey administered in native language of the subjects (S1 Table). Venous blood samples from children (~5 mL) and adults (~8 mL) were collected after enrollment and prior to commencement of anti-malarial treatment. An aliquot was transported to Noguchi Memorial Institute for Medical Research (NMIMR) and assessed by fluorescence-activated cell sorting (FACS). Plasma, red blood cells and buffy coats were obtained by centrifugation and stored at -80°C for later use.

#### Assessing Endothelial Progenitor Cell numbers and phenotype using FACS

Forty-two randomly selected samples were chosen for FACS analysis using a systematic sampling technique that picked every 12^th^ subject. Selection of EPC was based on dual positive CD34^+^CD309^+^ events [28]. The EPC population was defined as being CD45^-^CD34^+^CD309^+^ or CD45^-^CD34^+^CD133^+^_,_ to account for immature EPC [18]. EPC were analyzed as previously described [29]. Forward side scatter was used to eliminate debris and RBC. Gating strategy included; selection of CD45^-^ events from leukocyte Forward/Side Scatter dot plot, to exclude lymphoid cells, followed by gating for CD34^+^CD309^+^ or CD34^+^CD133^+^ double positive events for EPC quantification or CD34^+^CD284^+^ double positive events for EPC expression of TLR4 (S1 Fig). Aliquots of 200 μL of venous blood per reaction were incubated for 15 minutes in the dark with mouse anti-human phycoerythrin (PE)-conjugated or fluorescein isothiocyanate (FITC)-conjugated antibody pairs. EPC were isolated using specific antibody pairs; CD34-FITC and CD304-PE (VEGFR2-PE). To assess TLR, specific antibody pair CD34-FITC and CD284-PE (TLR4-PE) was used. Aliquots of cells incubated without antibodies or with appropriate isotype controls were used as controls. All antibodies were purchased from Miltenyi Biotec (Auburn, CA). After incubation, red blood cells were lysed with BD FACS lysing solution. Remaining leukocytes, which included EPC populations, were washed with BD FACSFlow solution, and immediately analyzed. Each analysis included 100,000 events, data compensation and analysis was performed using a BD FACScan System (BD Biosciences, San Diego, CA) and FlowJo (version 10.6).

#### Quantification of plasma Heme, CXCL10 and Heme Oxygenase-1

Plasma was centrifuged for 30 min at room temperature at 1200xg to remove contaminating red blood cells. Total heme was quantified using a colorimetric assay according to the manufacturer's instructions (BioAssay System, Hayward, CA).

To determine the relationship between malaria infection, plasma CXCL10 and HO-1 expression levels in malaria negative versus positive subjects and between free heme and CXCL10 *in vitro*, we examined plasma in patients and supernatants from HBVEC and CD34^+^-HSPC cell culture using commercially available Human CXCL10/IP-10 Quantikine ELISA kit (R&D Systems, Minneapolis, MN) and Human HO-1 Enzyme Immunoassay kit (ENZO Life Sciences, Plymouth Meeting, PA). CXCL10 and HO-1 levels were measured using optimal concentrations of standards and antibodies according to the manufacturer's instructions. The data was analyzed at 450 nm wavelength using a Spectra Max 190 fluorescence micro plate reader (Molecular Devices Corp., Sunnyvale, CA).

#### Cell Culture

Human brain microvascular endothelial cells (HBVEC) were cultured in endothelial basal media supplemented with 2% Fetal Bovine Serum and growth factors to obtain endothelial growth media (EGM-2, Lonza, Walkersville, MD). Human primary CD34^+^-HSPC were isolated from human bone marrow mononuclear cells and include both hematopoietic stem and progenitor cells (StemCell Technologies, Vancouver, Canada). CD34^+^-HSPC’s were isolated using immunomagnetic positive selection from human adult bone marrow. The cells were cultured in StemSpan SFEM II basal media supplemented with StemSpan 100 expansion cocktail, both from StemCell Technologies. Both HBVEC and CD34^+^-HSPC were passaged at 70–90% confluence, plated at a density of 2×10^5^ cells/mL and incubated at 37°C in 5% CO_2_ until ready for treatment.

#### TUNEL Assay

HBVEC and CD34^+^-HSPC were seeded at a density of 1×10^5^ cells/mL in 96-well plates. Fresh heme was prepared in 0.02 M NaOH. Cells were serum-starved for 4 hr, followed by exposure to 60 μM heme, vehicle (0.02 M NaOH) or positive control agent, 57 μM camptothecin, (CPT) for 18 hr. The Guava easyCyte flow cytometry system was used to quantify apoptosis with the TUNEL Kit for Flow Cytometry (Millipore, Billerica, MA). Cells were fixed and permeabilized using Guava TUNEL solution and apoptotic events were counted if they emitted a nucleated cell fluorescent signal, and exhibited the forward light scatter (FSC) intensity appropriate for a particle the size of a cell. Debris events with low FSC signal were not counted. All population events were analyzed using CytoSoft version 2.0 software. Data corresponds to three experiments run in parallel.

#### RNA Extraction and qRT-PCR

Total RNA was isolated from HBVEC and CD34^+^-HSPC using Qiagen RNeasy kit (Valencia, CA) and quantified using the Nanodrop N-1000 by Agilent Biosystems (Santa Clara, CA). The Qiagen QuantiTech Reverse Transcription kit was used to synthesize cDNA according to manufacturer instructions (Valencia, CA). The reverse transcription reactions were carried out in 20 μL volumes at 42°C for 15 min followed by 95°C for 3 min. TLR4 and CXCL10 expression were analyzed by quantitative RT-PCR and was performed using iQ SYBER Green Supermix (Bio-Rad laboratories, Hercules, CA) in 25 μL reaction volumes with glyceraldehyde 3-phosphate dehydrogenase (GAPDH) as control. The CFX96 Real-Time PCR System (Bio-Rad Laboratories, Hercules, CA) was used to perform qRT-PCR and analyzed the cycle threshold data obtained using thermocycling conditions: 95°C for 15 min, 95°C for 15 sec, 55°C for 30 sec, and 72°C for 30 sec for 40 cycles. Primer sequences were as follows; CXCL10: (FP 5’- TGACTCTAAGTGGCATTCAAGG, RP 5’-CAAAATTGGCTTGCAGGAAT), TLR4: (FP 5’- CAGGATGATGTCTGCCTCGC -3’, RP 5’- TTAGGAACCACCTCCACGCAG -3’), GAPDH: (FP 5’-GAAGGTGAAGGTCGGAGTC-3’, RP 5’-GAAGATGGTGARGGGATTTC-3’). The 2(-Delta Delta C(T)) method was used to analyze relative gene expression data normalized to the housekeeping gene, GAPDH. Results are expressed as fold change relative to housekeeping gene in treatment versus vehicle-treated cultures.

#### Statistical Analysis

Population distribution was determined using chi-squared test. Experiments were performed in triplicate and *p*-values were determined using T-test, Mann-Whitney U-test, ANOVA or two-way ANOVA and Tukey’s post hoc comparisons where appropriate. Data is presented as mean ± standard error or median and interquartile range (IQR) unless otherwise stated. A *p*-value < 0.05 was considered statistically significant. Statistical analyses were performed using GraphPad Prism version 6.0 for Windows (GraphPad Software, San Diego California USA).

## Results

A total of 575 participants were enrolled in the study; 147 non-malaria subjects and 428 malaria patients. There was a significant difference between the median age for non-malaria subjects (13 years, IQR 4–14 years) and malaria patients (5 years, IQR 2–8 years), *p* < 0.0001 (Table 1). There were no significant differences in gender between the two groups (*p* = 0.07, Table 1). Hematological indices showing significant differences between non-malaria and malaria subjects included Hemoglobin (non-malaria, 12.3 gm/dL vs malaria, 11.8 gm/dL, *p* < 0.0001), WBC (non-malaria, 7.1 gm/dL vs malaria, 6.0x10^3^/mL, *p* < 0.0001) and platelet (non-malaria, 235x10^3^/mL vs malaria, 146x10^3^/mL, *p* < 0.0001) (Table 1).

**Table 1:** Demographic and hematological characteristics.

Characteristic	Non-Malaria Median (IQR) N = 147	Malaria Median (IQR) N = 428	*p*-value
Age (year)	13 (4–14)	5 (2–8)	<0.0001
Gender (% male)	54.4%	46.7%	0.07
Hemoglobin (gm/dL)	12.3 (11.6–14.2)	11.8 (10.4–13.2)	<0.0001
WBC (x10^3^/mL)	7.1 (5.8–7.9)	6.0 (4.6–7.7)	<0.0001
Platelet (x10^3^/mL)	235 (162–303)	146 (97–197)	<0.0001
EPC Frequency*	0.3 (0.1–0.7)	0.12(0.1–0.2)	0.018

Dichotomous variables compared using χ^2^ and Fisher exact tests and continuous variables compared using Mann-Whitney tests. Values reported as percent and number of observations for dichotomous variables or median and Interquartile Range (IQR) for continuous variables. There was a significant difference in median age however the age ranges for both the Non-Malaria and Malaria groups were the same. There were no significant differences in sex of the participants in each group.

*42 samples were randomly selected for EPC analysis; non-malaria = 8, malaria = 34.

### *P*. *falciparum* infection decreases frequency of circulating EPC

**Fig 2:**
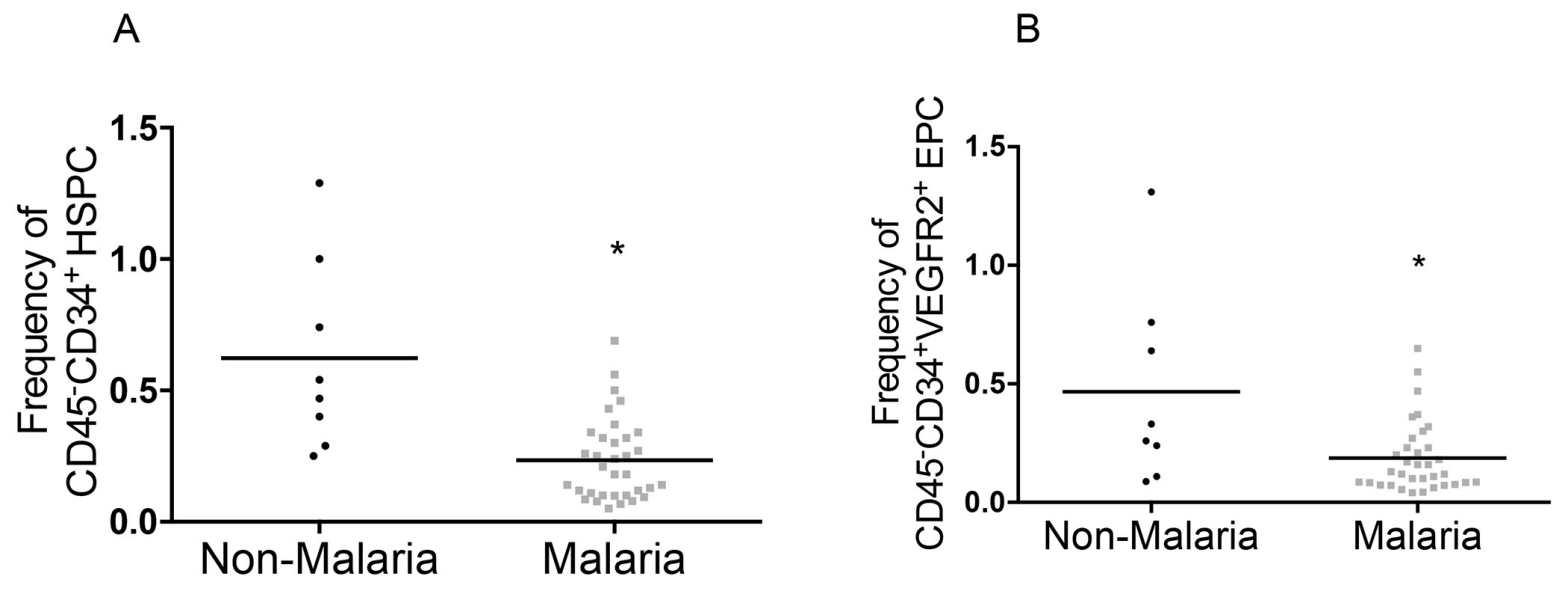
CD34^+^ cell populations decreased in malaria. (A) CD34^+^ hematopoietic stem and progenitor cell populations are significantly decreased in malaria patients. Median fluorescence intensity; 0.5 (IQR 0.3–0.9) in non-malaria, n = 8, vs. 0.2 (IQR 0.1–0.3) in malaria patients, n = 34, *p* = 0.0006. (B) Circulating EPC numbers (CD45^-^CD34^+^VEGFR2^+^) are decreased in malaria patients by 1.7-fold compared with non-malaria. Median fluorescence intensity; 0.3 (IQR 0.1–0.7) in non-malaria, n = 8 vs malaria 0.1(IQR 0.1–0.2), n = 34, *p* = 0.02. Data represented as median frequency and Interquartile Range (IQR). Mann-Whitney tests were used to calculate *p*-value.

To determine the effect of *P*. *falciparum* infection on circulating EPC populations, we measured frequency of EPC markers, both mature (CD45^-^CD34^+^VEGFR2^+^) and immature (CD34^+^VEGFR2^+^CD133^+^), in leukocyte fractions of whole blood from 42 randomly selected samples (8 non-malaria and 34 malaria) using FACS analysis. Randomization was accomplished using a systematic sampling technique that selected every 12^th^ subject for recruitment into the sub-study. The median frequencies of CD34^+^-HSPC and mature CD45^-^CD34^+^VEGFR2^+^-EPC were significantly decreased in malaria patients relative to non-malaria subjects; 0.5 (IQR 0.3–0.9) in non-malaria vs. 0.2 (0.1–0.3) in malaria patients, *p* = 0.0006 and 0.3 (IQR 0.1–0.7) in non-malaria vs. 0.1 (IQR 0.1–0.2) in malaria patients, *p* = 0.02 (Fig 2A and 2B. Immature EPC expressing CD34^+^VEGFR2^+^CD133^+^ are not reported due to very low numbers. Data are represented as the median numbers of EPC events per 100,000 leukocytes.

### *P*. *falciparum* infection is associated with increased plasma heme, HO-1 and CXCL10 levels

**Fig 3:**
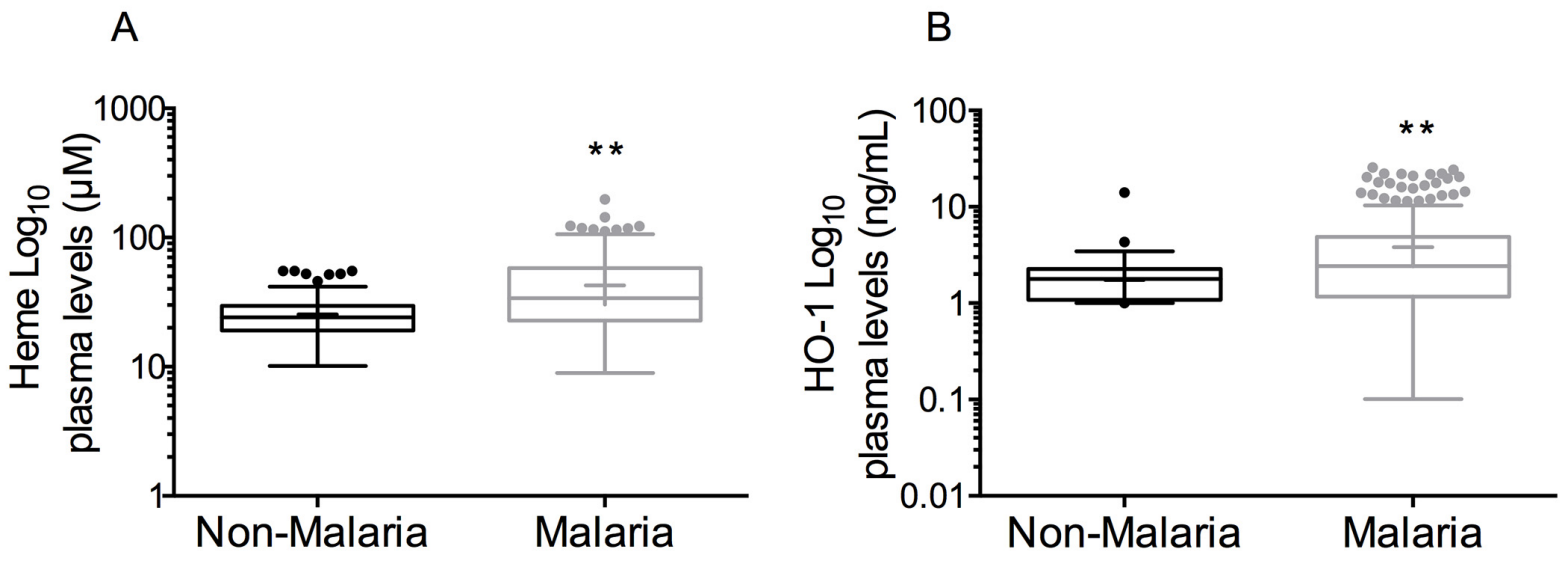
Plasma heme and HO-1 levels increase in malaria patients. (A) Malaria patients have increased expression of plasma heme (*p* < 0.0001) and (B) Heme Oxygenase-1 (HO-1) (*p* < 0.0001) compared to non-malaria subjects. Box plots representing medians with 25th and 75th percentiles, bars for 10th and 90th percentiles, and points for outliers of biomarker concentrations. Means indicated by (+) sign. Statistically significant *p*-values after Bonferroni adjustment are shown, n = 411. Normal range of 0–60 μM heme and 0–4 ng/mL HO-1 observed in non-malaria subjects.

**Fig 4:**
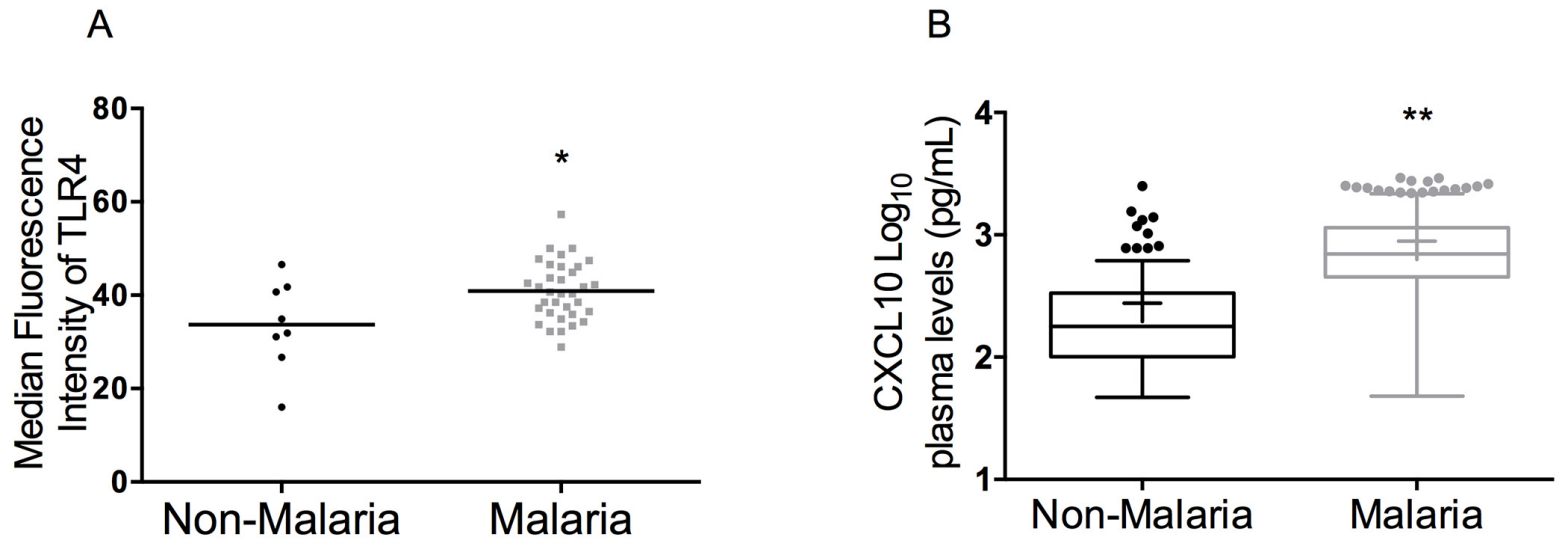
Malaria patients have increased expression of TLR4 and plasma CXCL10. (A) TLR4 Expression is increased in EPC of malaria patients: Median Fluorescence Intensity in non-malaria subjects, 33.7 vs malaria patients, 40.9, *p* = 0.04. The EPC population was defined as being CD45^-^CD34^+^CD309^+^, (non-malaria n = 8, malaria n = 34). (B) Plasma CXCL10 is significantly increased in malaria patients compared to non-malaria subjects (non-malaria subjects, 178.6 pg/mL (IQR 100.7–333.4), malaria patients, 698.3 pg/mL (IQR 453.8–1143), *p* < 0.0001). Normal range of 49–811 pg/mL in non-malaria subjects.

In 2012, Liu et al. reported significant increases in heme-induced HO-1 and CXCL10 in plasma in an experimental murine model of malaria and further confirmed their results *in vitro* [30]. This study and others demonstrated that free heme induces both HO-1 and CXCL10 expression *in vitro* and *in vivo*. To determine the levels of heme, HO-1 and CXCL10 in the plasma of malaria patients, chromogenic heme assay and HO-1 and CXCL10 immunoassays were performed. There were significant increases in plasma concentrations of heme non-malaria 24.1 μM (IQR 19.1–29.7), malaria 26.9 μM (IQR 20.1–39.5), *p* < 0.0001, Fig 3A), HO-1 (non-malaria 1.8 ng/mL (IQR 1.2–2.3), malaria 2.5 ng/mL (IQR 1.1–5.1), *p* < 0.0001, Fig 3B) and CXCL10 (non-malaria 180.4 pg/mL (IQR 101.1–328.6), malaria 705.7 pg/mL (IQR 459.0–1154), *p* < 0.0001, Fig 4B) among malaria patients compared to non-malaria subjects (Table 2).

**Table 2:** Plasma Heme, Heme-Oxyenase-1 and CXCL10 quantification.

	Non-Malaria N = 141*	Malaria N = 270†m	*p*-value
**Heme (μM)**			
All ages	24.1 (19. 1–29.7)	26.9 (20.1–39.5)	<0.0001
age ≤16 years	24.3 (19.1–29.7)	29.3 (21.5–46.1)	<0.0001
age >16 years	23.7 (16.3–29.4)	20.5 (17.9–25)	0.2
**Heme Oxygenase (ng/mL**)			
All ages	1.8 (1. 2–2.3)	2.5 (1. 1–5.1)	<0.0001
age ≤16 years	1.9 (1.4–2.6)	2.6 (1.1–5.5)	0.0006
age >16 years	0.4 (0.2–0.9)	2.4 (0.9–4.3)	<0.0001
**CXCL10 (pg/mL)**			
All ages	180.4 (101.1–328.6)	705.7 (459.0–1154)	<0.0001
age ≤16 years	198.1 (107.3–335.5)	666.2 (453.8–1024	<0.0001
age >16 years	121.6 (83.3–295.6)	1316 (534.4–1969)	<0.0001

Continuous variables compared using Mann-Whitney test, values reported as median and Interquartile Range (IQR). Malaria subjects had significantly higher levels of heme, HO-1 and CXCL10 compared with non-malaria regardless of the age group.

*Among the 141 non-malaria subjects 89% (125/141) are ≤16 years of age and 11% (16/141) are >16 years of age.

**†**Among the 270 malaria subjects 83% (223/270) are ≤16 years of age and 17% (47/270) are >16 years of age.

### *P*. *falciparum* infection increases expression of TLR4 in EPC and plasma CXCL10 *in vivo*

In 2012, Liu et al. reported significant increases in plasma heme induced HO-1 and CXCL10 in an experimental murine model of malaria and further confirmed their results *in vitro* [30]. This study and others demonstrate that free heme induces both HO-1 and CXCL10 expression both *in vitro* and *in vivo*. FACS analysis was used **t**o determine whether EPC exhibited altered expression of TLR4 in non-malaria versus malaria subjects. Median fluorescence intensity of TLR4 was assessed in 100,000 EPC events on a BD FACS Calibur. Statistical analysis of non-malaria versus malaria subsets indicated there was a significant increase in the median fluorescence intensity of TLR4 (CD284) expression on EPC in malaria patients (Fig 4A, non-malaria subjects, 33.7 vs malaria patients, 40.9, *p* = 0.04). These results confirmed previous reports indicating that pro-inflammatory factors, such as heme and CXCL10 are significantly associated with TLR4 expression in cells that modulate immunological responses [20, 31]. To determine the levels of CXCL10 in the plasma of malaria patients, CXCL10 immunoassays were performed. There were significant increases in median plasma concentrations of CXCL10 [non-malaria subjects, 178.6 pg/mL (IQR 100.7–333.4), malaria patients, 698.3 pg/mL (IQR 453.8–1143), *p* < 0.0001, Fig 4B]

### Heme induces apoptosis in human brain vascular endothelial and CD34^+^ hematopoietic stem and progenitor cells

**Fig 5:**
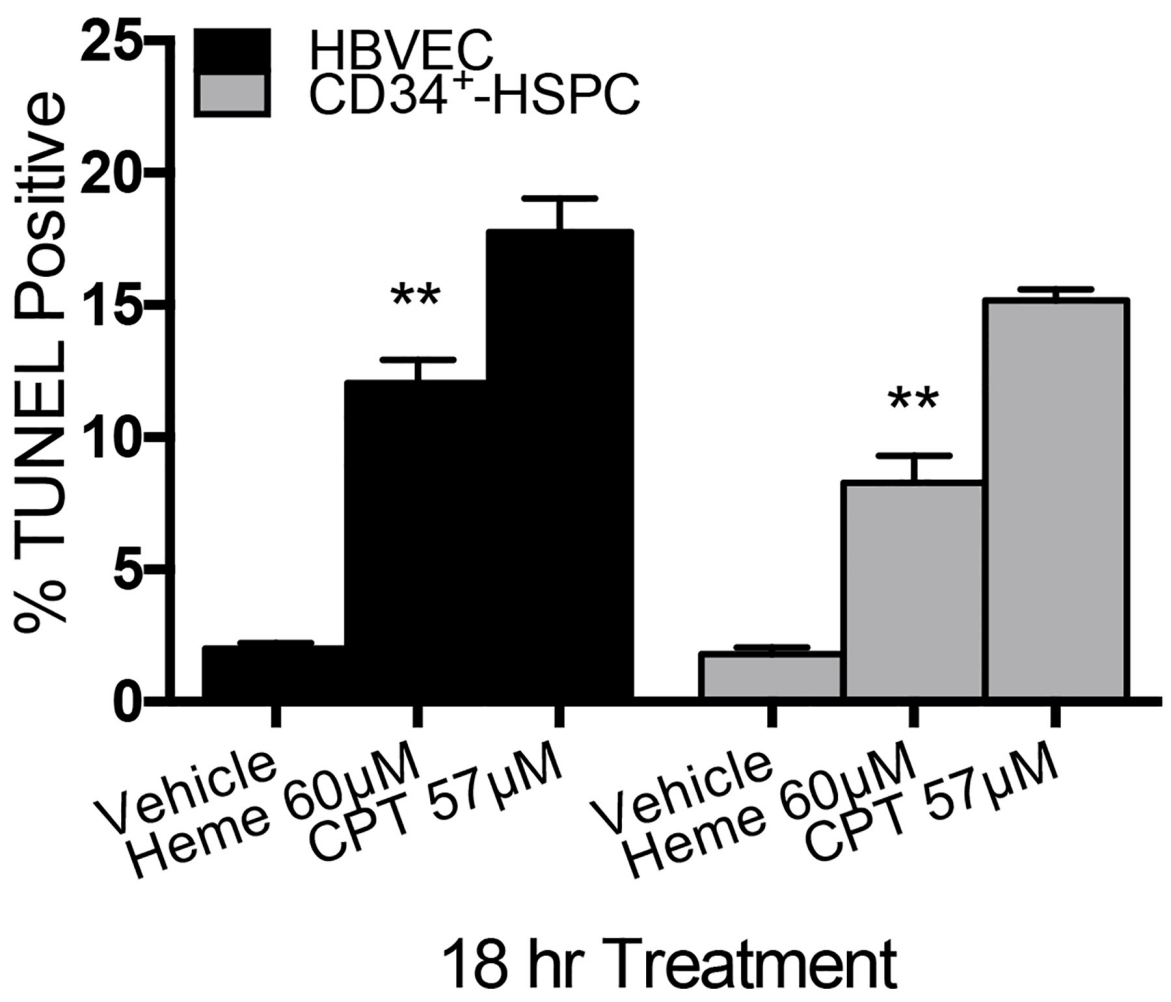
Heme induces apoptosis in HBVEC and CD34^+^-HSPC *in vitro*. Apoptosis was quantified using Guava TUNEL assay and analyzed by fluorescence-activated cell sorting (FACS). Apoptosis was analyzed using analysis of variance followed by Tukey’s multiple comparisons test; In HBVEC *p <* 0.0001 in heme-treated versus NaOH vehicle, and in CD34^+^-HSPC *p* = 0.0004 in heme-treated versus NaOH vehicle. CPT is a potent inducer of apoptosis used as positive control (*p* < 0.0001 in both cell types).

Previous studies demonstrated the functional role of free heme in human brain vascular endothelial cells (HBVEC) [8]. Here we confirm that heme (10–60 μM) decreases the viability of HBVEC and CD34^+^-HSPC in a concentration-dependent manner (Fig 5). A significant reduction in viability was noted after 18h treatment with 40 μM heme (*p* = 0.04 vs. vehicle) and the lethal dose for 50% (LD_50_) was obtained in both cell types with 60 μM heme, a physiologically relevant concentration in hemolytic diseases [32, 33]. This time point and concentration were used in subsequent experiments. Vehicle (0.02 M NaOH) did not significantly reduce cellular viability at any time. The TUNEL assay was used to determine whether a significant level of reduction in cell viability was due to apoptosis. Cells were treated with heme at LD_50_ doses and significant increases in apoptosis were observed (measured as percentage of TUNEL positive cells). Heme induced cell death via apoptosis in both HBVEC and CD34^+^-HSPC at 40 μM and 60 μM respectively (*p* = 0.04, Fig 5). 57 μM Camptothecin (CPT) was used as positive control.

### Heme-induced TLR4 activation in HBVEC and CD34^+^-HSPC

**Fig 6:**
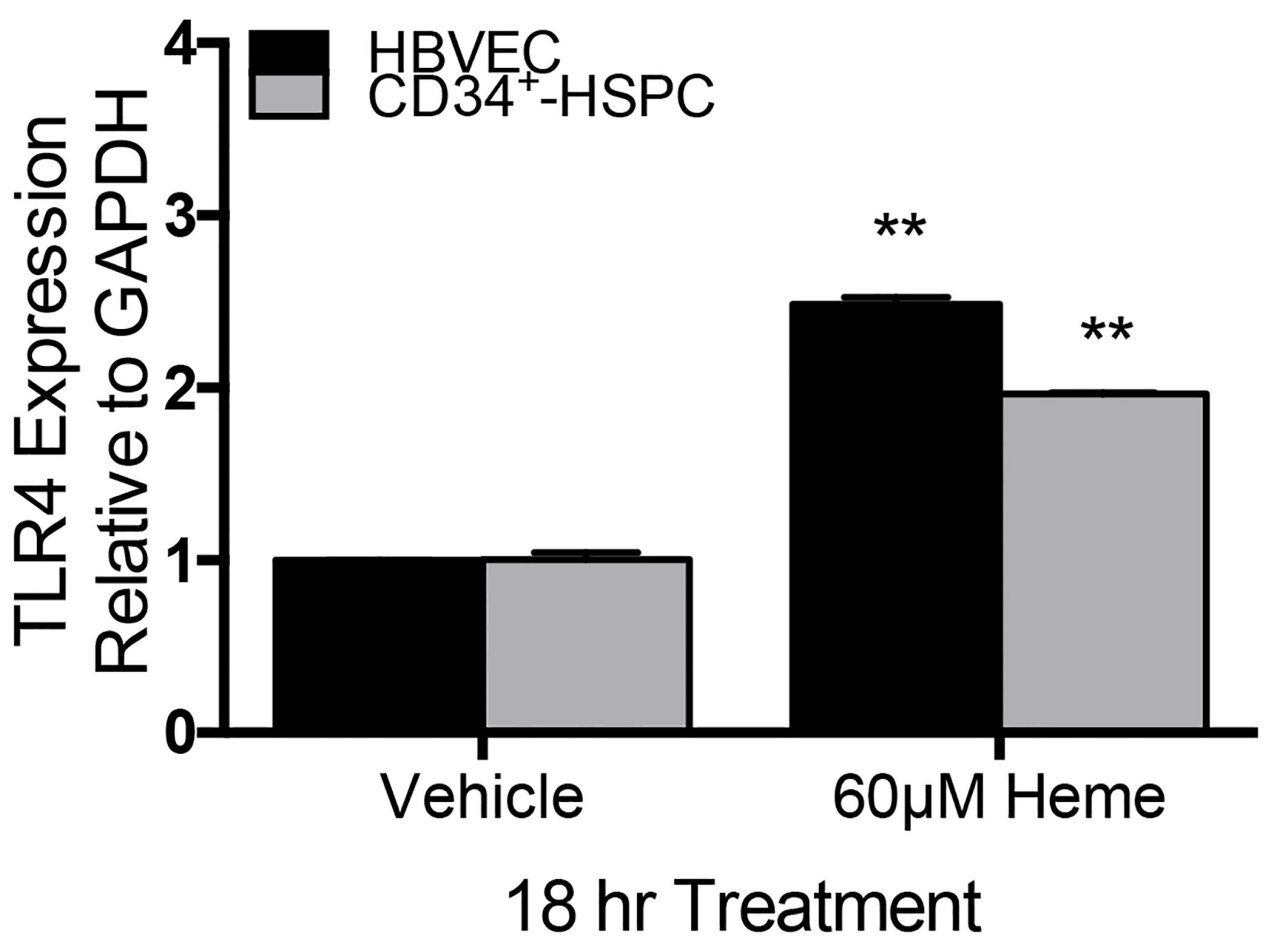
Heme mediates TLR expression *in vitro*. TLR4 mRNA expression was analyzed using student’s t-test. In HBVEC *p* = 0.002 in heme-treated versus NaOH vehicle and in CD34^+^-HSPC *p* = 0.005 in heme-treated versus NaOH vehicle. Data reported as mean fold-change relative to GAPDH.

Next we investigated the role of free heme in up-regulation of TLR4 mRNA in HBVEC and CD34^+^-HSPC *in vitro* using qRT-PCR. TLR4 mRNA was up-regulated in HBVEC and CD34^+^-HSPC when treated with heme at LD_50_ doses for 18 hr (Fig 6). TLR4 mRNA expression increased 2.5-fold in HBVEC and approximately 2-fold in CD34^+^-HSPC. Data was analyzed using student’s t-test; in HBVEC *p* = 0.002 and in CD34^+^-HSPC, *p* = 0.005. Having shown that TLR4 expression was modulated by exposure to heme, we tested the functionality of TLR4 in the presence of heme.

Heme-induced CXCL10 production in HBVEC and CD34^+^-HSPC is mediated by TLR4. To confirm that TLR4 mediates production of CXCL10 in microvascular and progenitor cells exposed to free heme, we assessed changes in CXCL10 protein and mRNA levels in HBVEC and CD34^+^-HSPC in the presence and absence of both a TLR4 signaling inhibitor, anti-CD14, and receptor binding antagonist, TAK-242, respectively. CD14 is a member of the LPS bacterial pattern recognition receptor (PRR) complex that physically associates with TLR4 and induces signal transduction and TAK-242 is a TLR4 antagonist that suppresses ligand-dependent and -independent signaling. Induction of CXCL10 mRNA expression by heme was assessed using qRT-PCR in both HBVEC and CD34^+^-HSPC. Expression of CXCL10 mRNA increased 7-fold in HBVEC and 2-fold in CD34^+^-HSPC when exposed to 60 μM heme for 18 hr, data was analyzed using analysis of variance followed by Tukey’s multiple comparisons test (Fig 6A). In HBVEC, *p <* 0.0001 in heme-treated cells compared with vehicle and *p* = 0.0003 in anti-CD14 plus heme treated cells compared to heme treated cells, and in CD34^+^-HSPC, *p* = 0.02 in heme-treated cells compared with vehicle and *p* = 0.11 in anti-CD14 plus heme treated cells compared to heme treated cells. Data is reported as mean fold-change relative to GAPDH ± SD. The data indicates that in the presence of free heme, HBVEC and CD34^+^-HSPC significantly increased their expression of CXCL10 mRNA, and blocking the extracellular domain of TLR successfully inhibits signal transduction leading to decreased CXCL10 mRNA expression in HBVEC.

**Fig 7:**
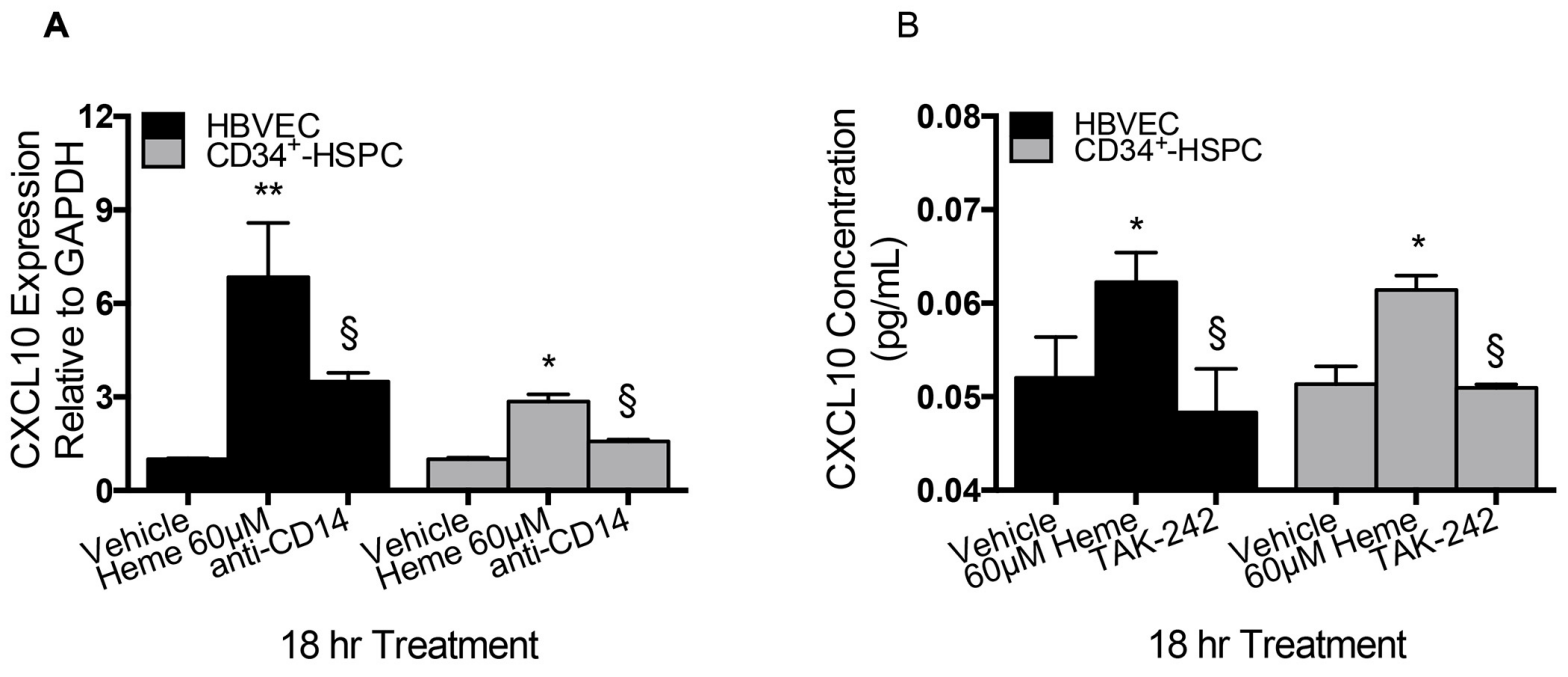
Heme-mediated stimulation of CXCL10 expression is TLR4 dependent. (A) CXCL10 mRNA expression is TLR4 dependent in HBVEC and CD34^+^-HSPC *in vitro*. CXCL10 mRNA expression was analyzed using analysis of variance followed by Tukey’s multiple comparisons test. In HBVEC and CD34^+^-HSPC, heme-treatment increased CXCL10 expression compared to vehicle (*p <* 0.0001 and 0.02, respectively). CXCL10 mRNA expression was decreased in the presence of anti-CD14 compared with heme alone (*p* = 0.0003 and *p* = 0.11, respectively). Data reported as mean fold-change relative to GAPDH. (B) CXCL10 expression is TLR4 dependent in supernatants of HBVEC and CD34^+^-HSPC *in vitro*. CXCL10 protein expression was analyzed using analysis of variance followed by Tukey’s multiple comparisons test. In HBVEC and CD34^+^-HSPC, heme-treatment increased CXCL10 expression compared to vehicle (*p* = 0.04 and 0.04, respectively). CXCL10 expression was decreased in the presence of TAK-242 compared with heme alone (*p* = 0.01, and 0.03, respectively). The 2(-Delta Delta C(T)) method was used to analysis relative gene expression data normalized to housekeeping gene, GAPDH, which was unaffected by experimental. Results are expressed as fold change relative to housekeeping gene in treatment versus vehicle-treated cultures.

CXCL10 protein expression is TLR4 dependent in supernatants of HBVEC and CD34^+^-HSPC *in vitro*. To determine whether the increase in CXCL10 protein expression was mediated through TLR4, we used TAK-242 to assess changes in expression of CXCL10 in the presence of 60 μM heme. HBVEC and CD34^+^-HSPC were treated with or without TAK-242 TLR4 antagonist for 1 hour prior to 18 hr heme treatment. CXCL10 protein expression was analyzed in 60 μM heme-treated versus vehicle-treated cells and TAK-242 plus 60 μM heme-treated versus vehicle-treated cells using analysis of variance followed by Tukey’s multiple comparisons test (Fig 7B). In HBVEC, *p* = 0.04 in heme-treated cells compared with vehicle and *p* = 0.01 in TAK-242 plus heme-treated cells compared to heme treated cells. In CD34^+^-HSPC, *p* = 0.04 in heme-treated cells compared with vehicle and *p* = 0.03 in TAK-242 plus heme-treated cells compared to heme treated cells. Data is reported as mean CXCL10 concentration ± SD. These data indicate that in the presence of free heme, HBVEC and CD34^+^-HSPC significantly increase expression of CXCL10 protein, and antagonist binding of TLR4 successfully inhibits signal transduction leading to decreased CXCL10 expression in HBVEC and CD34^+^-HSPC. This confirmed the functional role of TLR4 in heme-induced CXCL10 production.

## Discussion

We hypothesized that EPC depletion during malaria pathogenesis is a function of heme-induced apoptosis mediated by CXCL10 induction and Toll-like receptor (TLR) activation. In this study, individuals with malaria have significantly lower levels of EPC than observed in non-malaria. In addition, plasma levels of heme, heme oxygenase-1 (HO-1) and CXCL10 were significantly increased compared with non-malaria subjects.

We have previously shown that free heme is a potent apoptotic factor as well as inducer of the pro-inflammatory chemokine CXCL10 in microvasculature (HBVEC) *in vitro* and in murine models of experimental cerebral malaria (ECM) [11, 30]. Free heme in the plasma is generated during intravascular hemolysis when *Plasmodium* parasites scavenge erythrocytic hemoglobin. It has also been reported that free heme induces oxidative free radicals, leading to severe microvascular damage in sickle cell disease through induction of TLR4 [22, 34, 35]. Circulating endothelial progenitor cells play an important role in the repair and regeneration of damaged vascular endothelium as well as neovascularization in cerebrovascular disease [15, 36–40]. These cells are derived from CD34^+^ hematopoietic stem and progenitor cells, and these stem cell populations are depleted in individuals with severe malaria by an unknown mechanism [25, 41]. Heme is cytoprotective to human vascular endothelial cells at low concentrations when it induces the heme-neutralizing enzyme, HO-1, but is cytotoxic at very high concentrations [42–44]. Decreases in EPC were associated with increased heme and CXCL10 levels in addition to increased expression of TLR4 in malaria patients. To further establish the role of heme in malaria pathogenesis, we assessed the plasma levels of the heme-degrading enzyme HO-1 versus non-malaria subjects, and found that indeed they were increased as well. This suggests that in the pathogenesis of malaria, HO-1 has the potential to serve as an effective marker of heme toxicity and malaria severity.

The role of free heme in EPC depletion during severe malaria pathogenesis is poorly understood; therefore we explored the possibility that depletion of these endothelial cell precursors is mediated by TLR4 [19, 21, 45, 46]. In both human and murine malaria infection, increases in heme, CXCL10 and TLR4 and TLR9 have been shown to regulate the host immune response to *Plasmodium* infection [47–49]. In the present study, we have shown that heme-induced expression of CXCL10 and apoptosis was mediated by TLR4 in HBVEC and CD34^+^-HSPC. This confirmed *in vivo* observations of increased TLR4 expression in EPC populations in the presence of plasma heme and CXCL10 elevation. In addition, the TLR4 antagonist, TAK-242, dampened CXCL10 production *in vitro* in the presence of heme. Therefore, we propose a pathophysiological mechanism whereby heme mediates the TLR4 signaling pathway, resulting in overproduction of cytotoxic CXCL10. Thus depletion of EPC is a consequence of heme-induced CXCL10 production and TLR4-mediated apoptosis.

This study confirmed previous reports of the role of toll-like receptor activation in parasitic and inflammatory diseases. For example, glycosylphosphatidylinositol anchors from the protozoan parasite *Trypanasoma cruzi* parasites are potent activators of TLR2 in both mice and humans and prolonged exposure to low doses of TLR4 activating LPS decreases the repopulating potential of murine hematopoietic stem and progenitor cells and increases inflammatory cytokine production [21, 50, 51]. Additionally, bone marrow mononuclear and CD34^+^ cells from individuals with myelodysplastic syndromes, express increased levels of TLR4 when compared to the constitutive expression of TLR4 in the absence of these hematological syndromes [52]. It is also widely accepted, that in *P*. *falciparum* infection, there is a subset of individuals that have polymorphisms in TLR4 and TLR9 that have been linked to severe disease in both CM and ECM [47, 53, 54]. We have shown that integral components of the BBB, specifically HBVEC and CD34^+^-HSPC, are activated to increase expression of TLR4 in addition to excess CXCL10 production in the presence of increased heme, which mimics *in vivo* conditions in malaria. The expression of TLR4 by these populations makes them susceptible to the inflammatory effects of deleterious TLR4 activators TNFα and IFNγ, both of which are up-regulated in malaria [8, 20, 55]. In fact, knockout of MyD88, an adapter protein used by TLR4 to activate transcription factor NF-κB, resulted in decreased gene expression of these factors in the ECM mouse model [55, 56].

HBVEC showed significant reductions in expression of CXCL10 in the presence of the TLR4-blocking agent anti-CD14 *in vitro* conditions, though decreases in CXCL10 expression in CD34^+^-HSPC did not reach statistical significance. These findings indicate a resilience of CD34^+^-HSPC to receptor-mediated TLR4 signaling when compared to HBVEC. The high activation threshold in CD34^+^-HSPC may be cytoprotective and would explain why HBVEC had enhanced responses to heme, while CD34^+^-HSPC seemed to have dampened immunological response though susceptible to its apoptotic effects. Another possible explanation is that the high rate at which these cells undergo apoptosis in the presence of heme prevented detection of CXCL10 transcript in the presence or absence of TLR4 inhibitors.

Although this study assessed the role of heme in depleting EPC directly, another potential scenario is that mobilization of EPC from the bone marrow may be inhibited or inactivated in the presence of increased heme or other unknown cytotoxic factors [57, 58]. These circumstances would prevent detection of EPC populations using flow cytometric analysis of progenitor cell markers, a pitfall of this study. Lastly, expression of inflammatory receptors such as TLR4, would result in increased migration of EPC towards cites of localized endothelial damage, inducing activation, differentiation and potential sequestration of EPC from circulation, as reported elsewhere [20, 59]. Therefore, the intriguing question is whether EPC being depleted occurs due to cytotoxicity of free heme, sequestration or rapid activation and differentiation in the presence of increased serum CXCL10? We have shown that decreased bioavailability of EPC in malaria patients is due, in part, to heme toxicity, mediated by expression of TLR4 and further exacerbated by an exaggerated host inflammatory response due to excess production of CXCL10.

In this study we have shown that heme-induced, TLR4-mediated CXCL10 expression contributes significantly to depletion of HBVEC and CD34^+^-HSPC. Our results indicate that free heme is a major contributor to severe malaria pathophysiology by inducing apoptosis in HBVEC and CD34^+^-HSPC, which are vital BBB cellular components.

One aspect of malaria pathogenicity, the role of polymorphisms in human CXCL10, HO-1 and TLR genes have been reported but were not assessed here [47, 60–62]. In addition, age has been suggested to play a role in malaria pathogenesis, however, in our study age did not play a role in the expression of heme, CXCL10 and HO-1. These indices were higher in both adult and children malaria patients than non-malaria subjects of the same age group (Table 2). Therefore, the observed variations in host expression of these factors may be attributed to genetic variation inherent in sub-Saharan Africa. Further studies are underway to elucidate the precise mechanism(s) and/or genetic influences controlling EPC depletion in malaria in this geographic region.

Currently, there are few if any specific and sensitive biomarkers for prognosis of fatal malaria. Several host and parasite indicators of malaria infection have been identified as predictors of disease prognosis (from mild uncomplicated to severe malaria) including angiopoietins, elevated CXCL10 serum levels, CXCL10 polymorphisms and free heme [61, 63, 64]. Recently, many studies have investigated the use of EPC depletion as a potential biomarker for cancer and various inflammatory diseases, including malaria [65, 66]. Here we propose a TLR4-mediated role by which mature EPC are reduced in malaria patients. This study acknowledges that depletion of EPC is an important facet of malaria pathogenesis and identifies heme, CXCL10, TLR4 and circulating EPC levels as potential biomarkers for identification of individuals at risk of developing severe forms of the disease.

Heme induces production of apoptotic and inflammatory host factors, including CXCL10, and exacerbates malaria pathogenesis [11, 12]. Previous reports address the role of heme-induced CXCL10 in exacerbating severe malaria, and the vasculo-protective effects of HO-1’s ability to mediate CXCL10 expression [11, 43, 44, 67, 68]. Therefore potential adjunctive therapies would likely include bolstering HO-1 production or decreasing the amount of free heme in the plasma, thereby preventing the overexpression of angiostatic and inflammatory CXCL10 production. Another potential therapy would involve the use of hemopexin or haptoglobin in adjunctive therapies capable of quenching excessive free heme in malaria patients [69–72]. Lastly, blockage of TLR4 expression in brain microvasculature and circulating EPC would aide in decreasing the cytotoxic effects of heme by reducing its receptor-activated, apoptotic effects [20, 22]. In conclusion, this study has demonstrated that heme plays an important role in the viability of vital host cells such as HBVEC and EPC and should be further assessed in a wider range of populations and other brain microvascular supporting cell types for use in development of novel therapeutics in the prevention and treatment of severe malaria.

## Supporting Information

S1 Fig Gating strategy and selection of CD34^+^ cell populations from whole blood leukocyte fraction. The CD34^+^-HSPC population was defined as CD45^-^CD34^+^ and the EPC population was defined as CD45^-^CD34^+^CD309^+^ from Forward Scatter/Side Scatter upon elimination of debris and RBC. Gating strategy included selection of CD45^-^ events followed by gating for CD34^+^ or CD34^+^CD309^+^ double positive events.
(PDF)

S1 Table Human Subjects raw data sets. (XLSX)
