# Correction: Heme-Mediated Induction of CXCL10 and Depletion of CD34+ Progenitor Cells Is Toll-Like Receptor 4 Dependent

**DOI:** 10.1371/journal.pone.0147460

**Published:** 2016-01-14

**Authors:** Carmen M. Dickinson-Copeland, Nana O. Wilson, Mingli Liu, Adel Driss, Hassana Salifu, Andrew A. Adjei, Michael Wilson, Ben Gyan, Daniel Oduro, Kingsley Badu, Felix Botchway, Winston Anderson, Vincent Bond, Methode Bacanamwo, Shailesh Singh, Jonathan K. Stiles

In the Funding section, a grant number from the funder National Institute of Neurological Disorders and Stroke (NINDS) is missing. The additional grant number is: #1R56NS091616-01.
