# Supplementary material for: Heme-Mediated Induction of CXCL10 and Depletion of CD34+ Progenitor Cells Is Toll-Like Receptor 4 Dependent
Source: PLoS One. 2015 Nov 10;10(11):e0142328. doi: 10.1371/journal.pone.0142328 (PMC4640861; doi:10.1371/journal.pone.0142328)
Supplement: S1 Fig — The CD34+-HSPC population was defined as CD45-CD34+ and the EPC population was defined as CD45-CD34+CD309+ from Forward Scatter/Side Scatter upon elimination of debris and RBC. Gating strategy included selection of CD45- events followed by gating for CD34+ or CD34+CD309+ double positive events. (PDF) [file pone.0142328.s001.pdf]

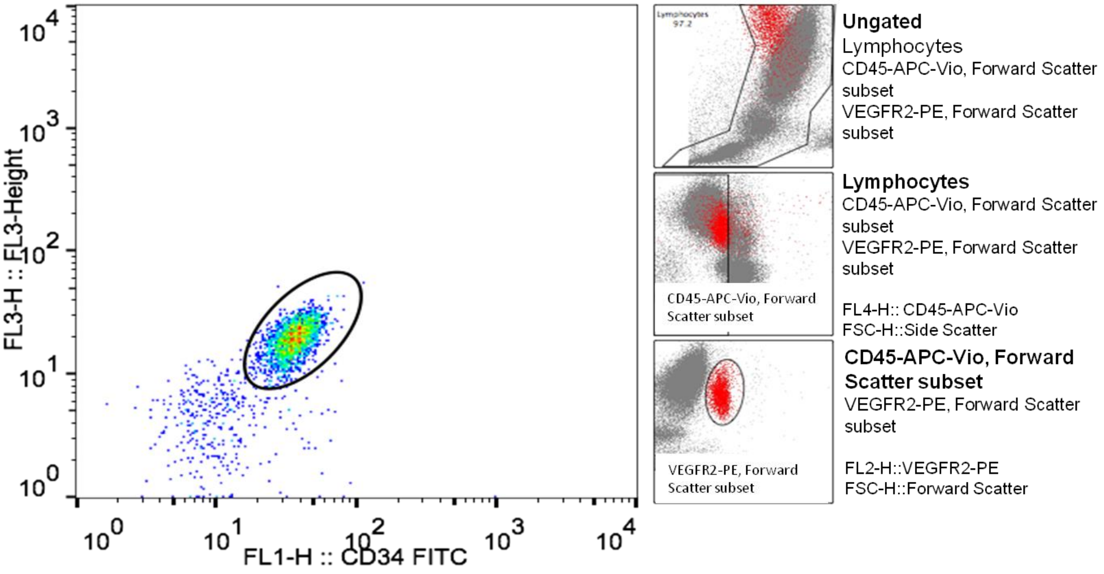
FL3-H :: FL3-Height
FL1-H :: CD34 FITC
10^0
10^1
10^2
10^3
10^4
Lymphocytes
97.2
CD45-APC-Vio, Forward Scatter subset
VEGFR2-PE, Forward Scatter subset
Ungated
Lymphocytes
CD45-APC-Vio, Forward Scatter subset
VEGFR2-PE, Forward Scatter subset
Lymphocytes
CD45-APC-Vio, Forward Scatter subset
VEGFR2-PE, Forward Scatter subset
FL4-H:: CD45-APC-Vio
FSC-H::Side Scatter
CD45-APC-Vio, Forward Scatter subset
VEGFR2-PE, Forward Scatter subset
FL2-H::VEGFR2-PE
FSC-H::Forward Scatter
